# Supplementary material for: The major subunit of widespread competence pili exhibits a novel and conserved type IV pilin fold
Source: J Biol Chem. 2020 Apr 9;295(19):6594–604. doi: 10.1074/jbc.RA120.013316 (PMC7212644; doi:10.1074/jbc.RA120.013316)
Supplement: Supporting Information [file supp_295_19_6594__index.html]

The major subunit of widespread competence pili exhibits a novel and conserved type IV pilin fold — ComGC pilin exhibits a novel type IV pilin fold — The major subunit of widespread competence pili exhibits a novel and conserved type IV pilin fold — ComGC pilin exhibits a novel type IV pilin fold — Supporting Information 

# The major subunit of widespread competence pili exhibits a novel and conserved type IV pilin fold

## Supporting Information

- Supporting Information (to be published online) - Supporting Tables and Figures
- Supporting Information (to be published online) - Supporting Spreadsheet 1
- Supporting Information (to be published online) - Supporting Spreadsheet 2
