# Supplementary material for: The major subunit of widespread competence pili exhibits a novel and conserved type IV pilin fold
Source: J Biol Chem. 2020 Apr 9;295(19):6594–604. doi: 10.1074/jbc.RA120.013316 (PMC7212644; doi:10.1074/jbc.RA120.013316)
Supplement: Supporting Information [file supp_RA120.013316_159266_3_supp_506614_q88sbr.pdf]

The major subunit of widespread competence pili exhibits a novel and conserved type IV pilin fold

Devon Sheppard[1], Jamie-Lee Berry[1], Rémi Denise[2,3], Eduardo P. C. Rocha[2], Steve Matthews[4], Vladimir Pelicic[1]*

From the [1]Medical Research Council Centre for Molecular Bacteriology and Infection, Imperial College London, London SW7 2AZ, United Kingdom; [2]Microbial Evolutionary Genomics, Institut Pasteur, CNRS, UMR3525, Paris 75015, France; [3]Sorbonne Université, Collège doctoral, Paris 75005, France; [4]Centre for Structural Biology, Imperial College London, London SW7 2AZ, United Kingdom

*To whom correspondence should be addressed: Vladimir Pelicic: Imperial College London, Medical Research Council Centre for Molecular Bacteriology and Infection, London SW7 2AZ, United Kingdom; v.pelicic@imperial.ac.uk; +44 20 7594 2080.

Table S1. Bioinformatic analysis of the components of *S. sanguinis* Com machinery.

**Name**	**MW (Da)***	**pI***	**InterPro domain**	**predicted function**	**identity in *B. subtilis* (%)***	**identity in *S. pneumoniae* (%)***
ComGA	35,864	6.17	IPR001482	extension ATPase	33.01	74.76
ComGB	37,817	9.06	IPR003004	platform protein	21.36	67.37
ComGC	10,184	7.70	IPR016940	major pilin	33.33	65.59
ComGD	14,680	6.40	-	minor pilin	20.66	55.04
ComGE	9,689	10.17	IPR021749	minor pilin	13.92	52.33
ComGF	15,787	5.25	IPR016977	minor pilin	24.41	61.76
ComGG	13,663	9.32	-	minor pilin	19.80	38.84
ComC	24,816	9.23	IPR010627	prepilin peptidase	24.20	57.53

*For the pilins, the numbers correspond to the processed proteins.

Table S2. Plasmids used in this study.

Name	Description	Source
pET28-b	T7-based expression vector	Novagen
pMA-T-*comGC*$_{SS}$	pMA-T-derivative with codon-optimised *comGC*$_{SS}$	this study
pMA-T-*comGC*$_{SP}$	pMA-T-derivative with codon-optimised *comGC*$_{SP}$	this study
pET28-*comGC*$_{SS}$	pET28-derivative for expressing His$_6$-ComGC$_{SS}$	this study
pET28-comGC$_{SS}$	pET28-derivative for expressing His$_6$-ComGC$_{SP}$	this study

Table S3. Primers used in this study.

Name	Sequence*
comGC$_{SS}$-F	ggg**ccatgg**atcatcatcatcatcatcatAATCTGACCAAACAGAAAGATGCAGTTAGC
comGC$_{SS}$-R	ccc**ggatcc**TTAATTTGCAACGGTCTGGGTTTCACCG
comGC$_{SP}$-F	ggg**ccatgg**atcatcatcatcatcatcatAATCTGACCAAACAGAAAGAAG
comGC$_{SP}$-R	ccc**ggatcc**TTAATCGTTCACTTTGCGATTGGCACC

*Overhangs are in lower case. Restriction sites are in bold.

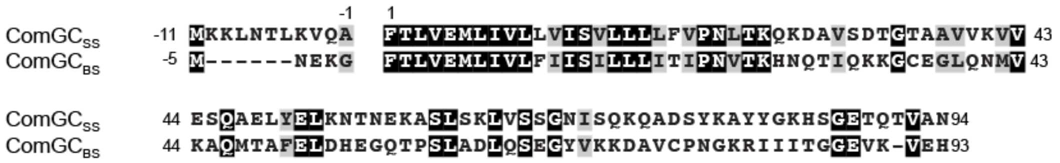

Fig. S1. Sequence alignment of ComGC in *S. sanguinis* and *B. subtilis.* Residues were shaded in black (identical), grey (conserved) or unshaded (different). The leader peptide, which is processed by the prepilin peptidase ComC is highlighted.

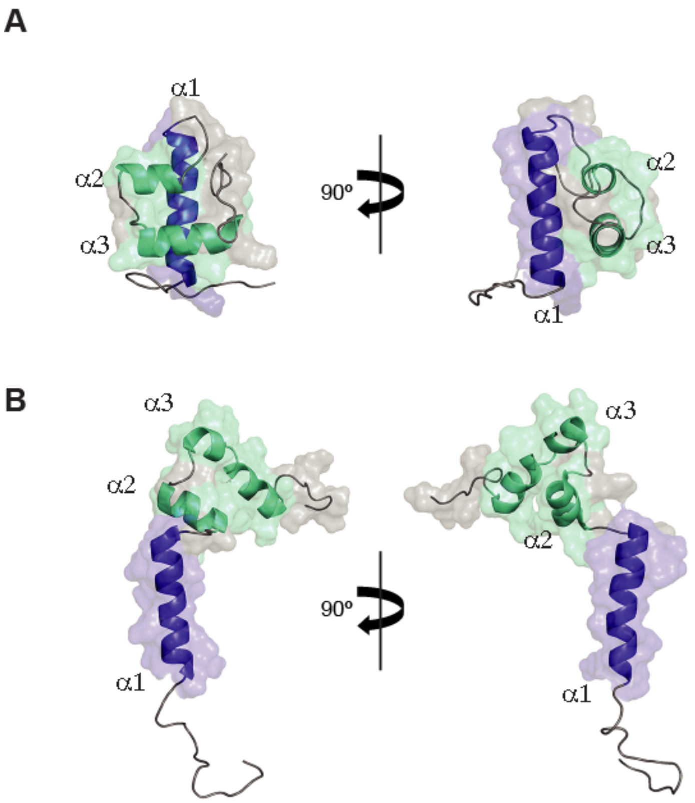

Fig. S2. Our ComGC$_{SP}$ structure differs markedly from a previously published one (PDB 5NCA). Cartoon representations of the two averaged solution structures, face and side views, are shown. We used the same nomenclature and colour scheme than in Fig. 4 and Fig. 5. (**A**) Our ComGC$_{SP}$ structure. (**B**) PDB 5NCA ComGC$_{SP}$ structure.

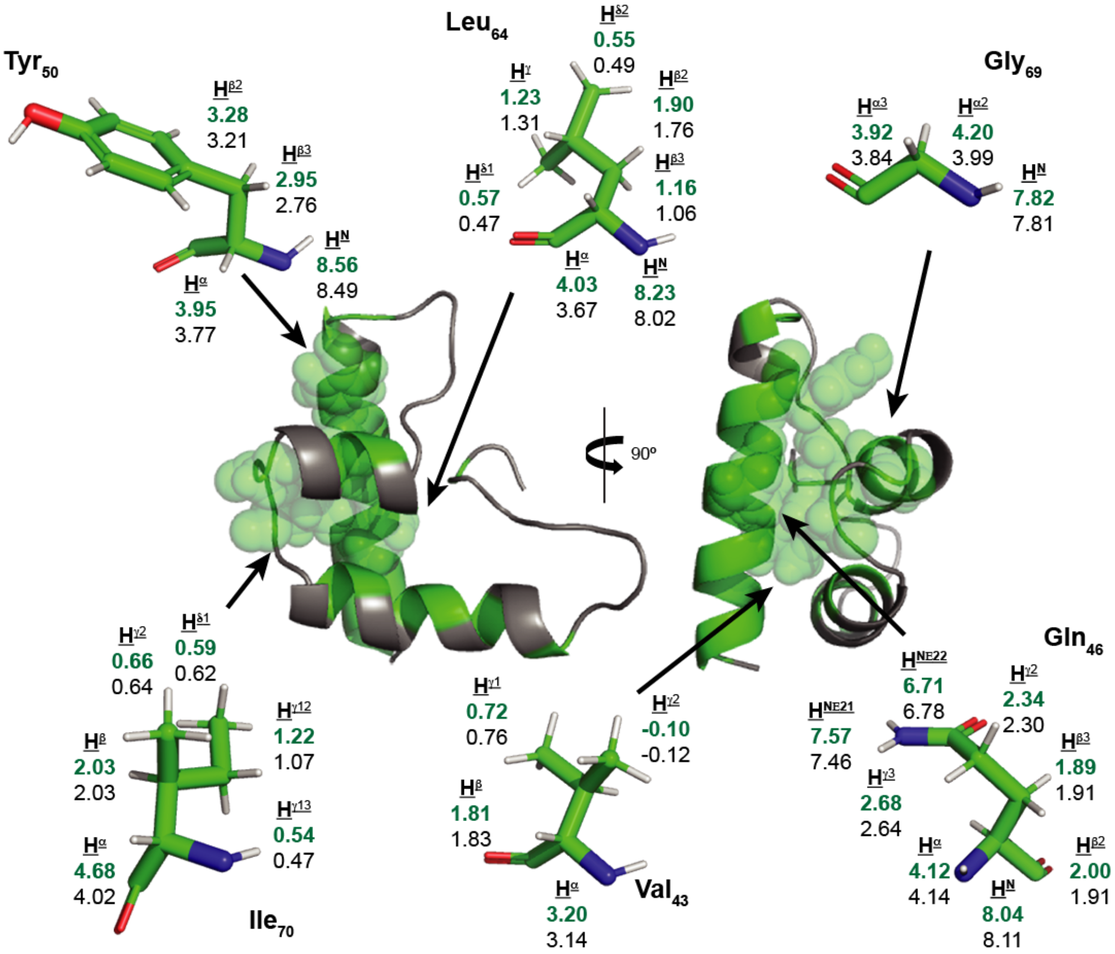

Fig S3. Conserved residues contributing to the globular fold of ComGC show unusually high chemical shift conservation between $ComGC_{SS}$ and $ComGC_{SP}$ structures. Cartoon representation of the ordered residues of $ComGC_{SS}$, where conserved residues are highlighted in green, with residues Val_{43}, Gln_{46}, Tyr_{50}, Leu_{64}, Gly_{68} and Ile_{70} shown with space filling representation. For these residues, ^{1}H chemical shifts are shown for $ComGC_{SS}$ (top in bold green) and $ComGC_{SP}$ (bottom).

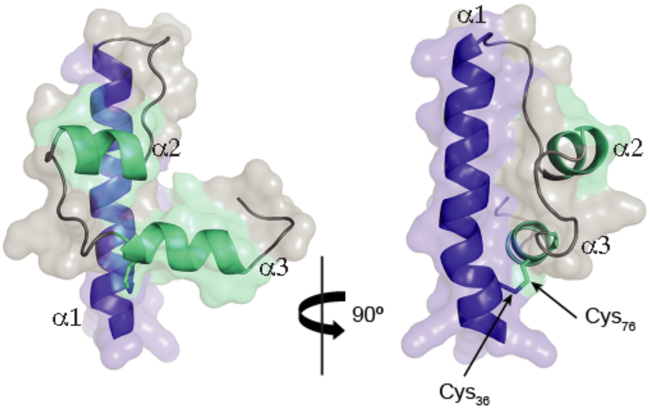

Fig. S4. 3D model of the globular ComGC$_{BS}$ identifies the disulfide bond known to stabilise that pilin. ComGC$_{SS}$ has been used as a template to generate a structural model of the globular head of ComGC$_{BS}$. The two cysteines known to form a disulfide bond, Cys$_{36}$ and Cys$_{76}$, are found in close proximity and are expected to significantly stabilise the globular fold.

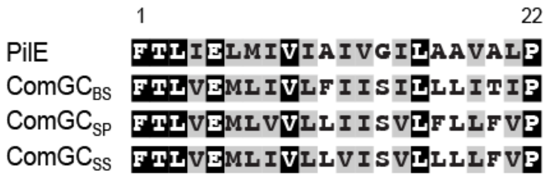

Fig. S5. The N-terminus in ComGC pilins is highly similar to that of other bacterial T4F major pilins. Sequence alignment of the N-termini of processed ComGC$_{SS}$, ComGC$_{SP}$, ComGC$_{BS}$, and PilE from *N. gonorrhoeae* T4P. Residues were shaded in black (identical), grey (conserved) or unshaded (different).

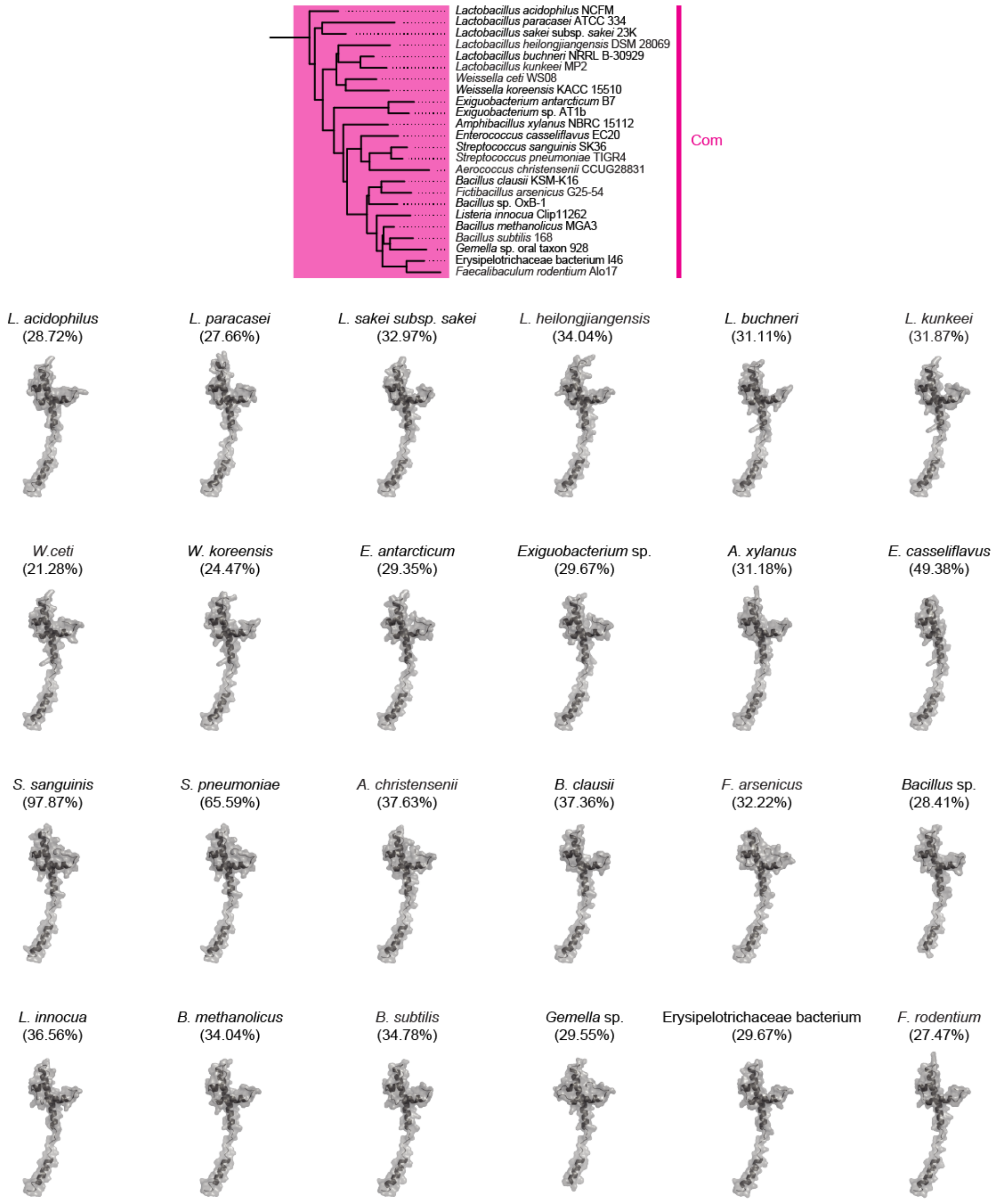

Fig. S6. Full-length 3D models of ComGC from species used to generate the phylogenetic tree in Fig. 3. The 3D structures have been modelled using as template (i) our averaged *S sanguinis* ComGC$_{SS}$ structure for the globular head, and (ii) the PulG pilin from *K. oxytoca* for the protruding α1N.

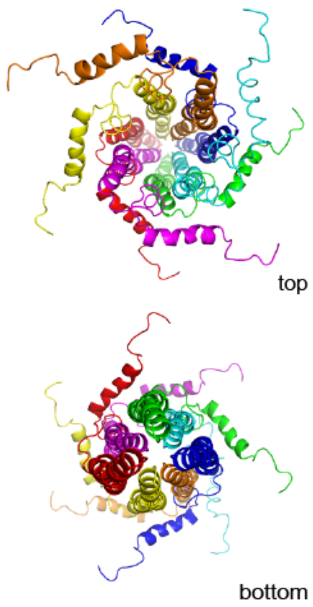

Fig. S7. 3D model of ComGC pili using the previously published (PDB 5NCA) ComGC$_{SP}$ structure. Introducing the previous ComGC$_{SP}$ structure into our SWISS-MODEL of ComGC filaments shows a high degree of steric clashes.

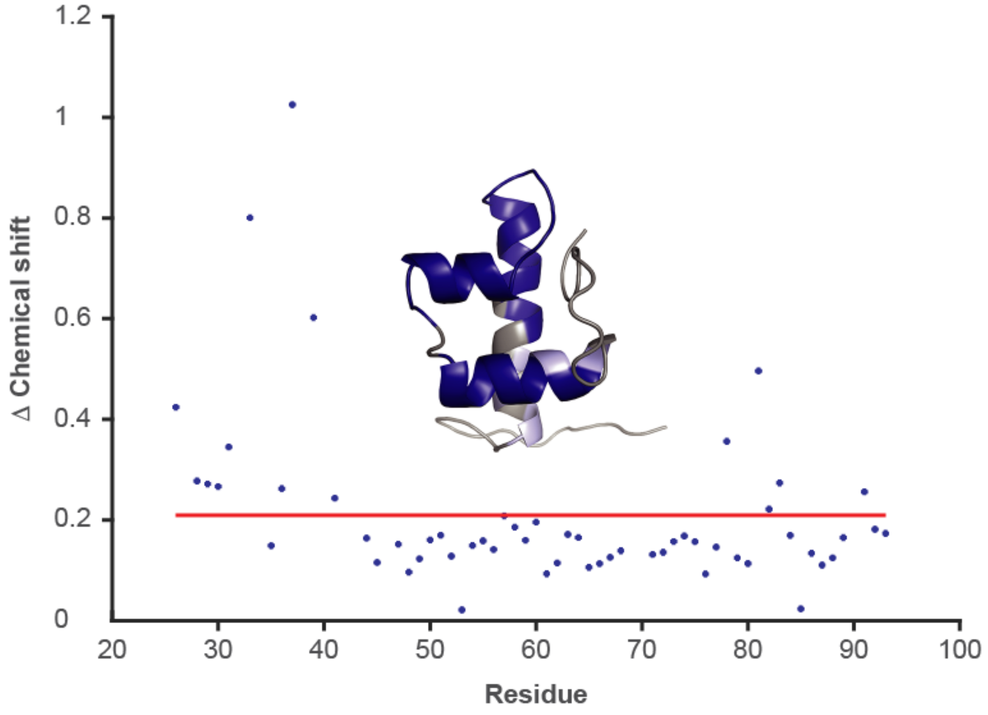

Fig S8. Amide chemical shift differences between our ComGC$_{SP}$ structure and the previously published one (PDB 5NCA). Amide chemical shift differences [ω_H+(γ_N/γ_H)ω_N] are presented against residue number. The average chemical shift difference is indicated as a red line. Those residues for which the difference in chemical shift is greater than the average ± standard deviation are highlighted onto our averaged ComGC$_{SP}$ structure in light blue (inset).

Supplemental Spreadsheet 1. List of all the Com systems detected by a search of the RefSeq database (from April 2019) using MacSyFinder with the corresponding Com model.

Supplemental Spreadsheet 2. List of all the species that have the potential to express a Com pilus.
